# Choice of Flap Affects Fistula Rate after Salvage Laryngopharyngectomy

**DOI:** 10.1038/srep09180

**Published:** 2015-03-17

**Authors:** Huang-Kai Kao, Mohamed Abdelrahman, Kai-Ping Chang, Chao-Min Wu, Shao-Yu Hung, Victor Bong-Hang Shyu

**Affiliations:** 1 Department of Plastic and Reconstructive Surgery, Chang Gung Memorial Hospital, Chang Gung University College of Medicine, Tao-Yuan, Taiwan; 2 Department of Otolaryngology-Head & Neck Surgery, Chang Gung Memorial Hospital, Chang Gung University College of Medicine, Tao-Yuan, Taiwan

## Abstract

Due to the significant morbidity and mortality associated with pharyngocutaneous fistula in pharyngoesophageal reconstruction following cancer resection, the purpose of this retrospective study is to examine the selection of tubed skin flaps that impact anastomotic integrity. The flaps evaluated included radial forearm flap versus anterolateral thigh flap, and fasciocutaneous anterolateral thigh flap versus chimeric anterolateral thigh flap. The outcome of interest is the incidence of pharyngocutaneous fistula. The radial forearm group had a significantly higher rate of fistula than the anterolateral thigh group (56.6% vs. 30.2%, *p* = 0.03). No significant difference in the incidence of fistula was demonstrated between fasciocutaneous and chimeric anterolateral thigh flap (36.8% vs. 25%, *p* = 0.51). The anastomotic integrity in pharyngoesopharyngeal reconstruction is affected by choice of skin flaps. Anterolateral thigh flap appears to be a viable option for pharyngoesophageal reconstruction. The more technical demand of the anterolateral thigh flap must be weighed against an easily harvested radial forearm flap.

Reconstruction of the esophageal defect is one of the most challenges for reconstructive surgeons. The advent of microsurgical techniques and advances in understanding of flap surgery have allowed for the successful reconstruction of complex defects involving the hypopharynx, cervical esophagus, and voice mechanisms in a single-stage operation with minimal complications.

Accepted reconstructive modalities include anterolateral thigh (ALT) flap, radial forearm (RF) flaps, jejunal flap, gastro-omental flap, and pectoralis flap. The choice of flap depends on individual surgeon's expertise and preference. A consensus on the flap selection has not been reached^1^,^2^,^3^,^4^. Reconstruction using the jejunum has the advantage of providing active transport of food by peristalsis. However, the morbidities associated with open abdominal surgery, such as postoperative ileus, risk of bowel adhesion and obstruction, incisional hernia, and pulmonary embarrassment are still the major concern. Our favorable and rather extensive experience with radial forearm flap and ALT flap in head and neck reconstruction led us to use these versatile flaps for pharyngoesophageal reconstruction^5^. In many units these flaps have been used in preference to enteric free flap reconstructions^6^,^7^,^8^.

The versatile use of the free radial forearm flap for pharyngoesophageal reconstruction has been proven useful in previous studies, due to its thin and pliable properties and consistent vascular pedicle. However, it has the drawbacks of cosmetic and functional donor-site morbidity, limitation in flap size and volume, the sacrifice of one major artery, and exposure of tendon due to partial skin graft loss^9^,^10^,^11^.

Since 2000, with the development and popularity of perforator flaps, the ALT flap has become the most prevalent choice for head and neck reconstruction in our institution. The advantages of ALT flap include consistent and reliable anatomy, long vascular pedicle, being far from the ablative site and allowing a two-team approach, feasibility to create multiple skin paddles by recruiting additional perforators, flexibility to reconstruct composite defects by recruiting different tissue types (adipose, muscle, and fascial components) all based on a single pedicle, and low donor site morbidity^6^. In pharyngoesophageal reconstruction, the tubed free ALT flap appears to offer better speech and swallowing functions and quicker recovery, and is more cost-effective than the jejunal flap^12^,^13^,^14^,^15^,^16^,^17^.

Pharyngocutaneous fistula in particular is an important early post-operative complication that results in reoperation, delayed discharge, surgical site infection, and psychological and financial distress. The purpose of this retrospective study is to examine the selection of tubed skin flaps that impact anastomotic integrity following pharyngoesophageal reconstruction. Through this analysis, we aim to gain a greater understanding of the collective impact of these parameters and their contribution to anastomosis failure.

## Results

There were 73 patients enrolled in the study. Patients' demographics and clinical details of the two groups are summarized and compared in Table 1. The mean age of the RFF group was 55.1 years (range: 39–75) and that of the ALT group was 56.6 years (range: 36–82). There were no significant differences in tumor stage and location. Regarding the operative variables, there was no significant difference when comparing the operation time, defect type, used flap size, and flap loss rate. Primary closure of the donor site was achieved in 34 patients (79.1%) of the ALT group. However, skin graft was required to close the donor site defect in all patients of the RFF group. Furthermore, the RFF group had a significantly prolonged hospital stay (*p* = 0.03) compared to patients in the ALT group.

Table 2 shows the comparison of clinical details between the fasciocutaneous ALT skin tube and the chimeric ALT skin tube. There were no differences in tumor stage, location, operation time, defect type and type of donor site closure. The fasciocutaneous ALT group had a significantly longer hospital stay (*p* = 0.04) than the chimeric ALT group.

Recipient site complications, medical site complications, and post-operative diet are listed in Table 3. The rate of stricture was 50.0%, 36.8%, and 20.8% in the RFF, fasciocutaneous ALT, and chimeric ALT flap groups respectively. One case of the RFF group and two cases of the ALT flap group developed carotid blowout. All of them were successfully treated with vessel ligation without neurologic sequelae. Swallowing function was determined by the highest level of diet achieved after surgery. Two patients were excluded from diet evaluation because of early death, including one patient in the RFF group and one patient in the ALT flap group. Among them, 26 patients (89.6%) in the RFF group and 37 patients (88.1%) in the ALT flap group achieved oral intake but with no significant difference.

Pharyngocutaneous fistula following reconstruction, which remains a dreaded postoperative complication, was compared and listed in Table 4. The overall fistula rates were 30.2% and 56.6% for the ALT and RFF group, respectively, and this difference achieved statistical significance (*p* = 0.03). There was no difference between the fasciocutaneous and chimeric ALT group.

## Discussion

In spite of advances in head and neck microsurgical reconstruction, pharyngocutaneous fistula remains a persistent and serious obstacle for the surgeons, because it has significant impact on the hospitalization length, permanent sequelae, functional status, and quality of life. The success of an esophageal anastomosis therefore depends upon meticulous attention to detail and the optimization of factors in two domains. First, patient-related systemic variables can influence anastomotic integrity, including pre-existing medical disease, nutritional status, and tumor stages. Second, the technical preparation requires precise flap choice and dissection, tailored flap design, and a tension-free anastomosis.

The workhorse free fasciocutaneous flaps used in pharngoesophageal reconstruction are anterolateral thigh and radial forearm flaps. The radial forearm flap is certainly the easiest as its long pedicle, rich blood supply, and tolerance to a longer period of ischemia makes it a surgeon friendly flap. Skin islands of 12–14 cm in length can be harvested without problem^19^,^20^. Nevertheless, there are many potential side-effects, including delays in wound healing because of partial or total loss of the skin graft, tendon exposure with ensuing stiffness and poor aesthetic outcomes, minimal decrease of hand function because of hypovascularization (cold intolerance), and transient or permanent numbness of the first two fingers and dorsum of the hand. However, no significant functional loss was observed in the literature^18^.

ALT has become the first-line reconstructive option for soft tissue defects because of its anatomic consistency, reliability, and plastic versatility. Flap-related perioperative mortality is almost nonexistent, and donor-site morbidity is one of the lowest of any pedicled or free flap reconstructive options available. ALT flap also excels over jejunum in producing superior tracheoesophageal speech while providing similar fistula and stricture rates, as demonstrated by Yu et al^6^. Moreover, in selected patients, extensive portions of the vastus lateralis muscle can be taken together with the skin paddle or as an independent portion of a chimeric flap. This will further protect the suture line and cover the great vessels in the neck after reconstructive surgery^20^,^21^,^22^. For patients with complex pharyngoesophageal, tracheal, and anterior neck defects, the ALT flap, which can be divided into two skin islands between two cutaneous perforators, could provide one-stage reconstruction without the need of a second flap.

Because of the above, we advocate the use of ALT as it can provides larger amount of tissue for reconstruction with less donor site morbidity than RFF. Our institution has shifted practice to the use of ALT flap as the primary flap for pharyngoesophageal reconstruction since 2000, instead of radial forearm flap^4^.

In this study we reviewed the technical factors that might lead to postoperative fistula after pharyngoesophageal reconstruction. The first consideration is the type of the free flap used, and our results match previous data that supports the superiority of the outcomes of pharyngoesophageal reconstruction using ALT flap over the RFF^4^. In our study, 30.2% of our patients developed a pharyngocutaneous fistula when using ALT flap, in comparison to 56.6% of patients using RFF, which represents a significant difference. We attribute this to the abundance and diversity of tissues provided by ALT flap, which allows surgeon to use many of the modified anastomotic techniques described by many authors to avoid anastomotic problems^23^,^24^,^25^. These extra tissues, such as the generous amount of fascia lata safely harvested from the donor site, can be used to wrap the first-layer suture. Additionally, portions of the vastus lateralis muscle can be taken together with the skin paddle or as an independent portion of a chimeric flap. In this study, patients with an ALT flap had shorter hospital stays (37.2 ± 12.5 days) in comparison with RFF group (48.6 ± 15.8 days), which represents a significant difference. These outcomes suggest that patients reconstructed with an ALT flap tend to recover more quickly postoperatively and have shorter hospital stays, similar to previous studies of the same kind^17^.

The reported incidence of pharyngocutaneous fistula has been very variable reaching as high as 65% in the literature. Tsou et al. found preoperative CCRT to be an independent risk factor that significantly raised the risk of fistula formation, from 21.4% in 112 patients receiving primary total laryngopharyngectomy and reconstruction to 58.3% in 48 patients undergoing post-irradiated salvage laryngopharyngectomy and reconstruction^26^. According to Yu's study, the incidence of fistula using ALT skin tube for pharyngoesophageal reconstruction was 9%. There was no significant difference in the rate of fistula between patients with or without a history of radiotherapy^3^. In this study, the rate of fistula in the radial forearm group or the ALT group was relatively higher when compared with other studies. This was likely all patients in the study already had disease recurrence after concurrent chemoradiotherapy (CCRT). Due to pre-operative CCRT, cancer recurrence, and aggressive salvage surgery, the tissue's capacity to heal was challenged to its limits.

The critical points to prevent pharyngocutaneous fistula after pharyngoesophageal reconstruction are the creation of a graft with adequate length and sufficient blood supply. Any degree of tension will interfere with the blood circulation and contribute to leak, fistula formation, or contracture with stricture at the anastomosis. The skin territory provided by the ALT flap is big and reliable, as one perforator can safely supply an area of up to 9 cm of skin paddle; this territory further increases with the number of perforators included. The RFF can provide a skin territory of up to 10 cm, but as the size of the flap increases, the blood supply in the distal parts decreases. Additionally, the blood supply will compromise more when the flap is folded for reconstruction, decreasing the perfusion and leading to leaks and future strictures in the case of RFF^27^.

The ALT flap is criticized sometimes for being very bulky for some types of reconstruction, while the RFF provides a very thin skin component that helps in ease of design. Nevertheless, due to the burden of malignancy and starvation, head and neck cancer patients do loose a reasonable portion of the donor site subcutaneous fat, which makes the ALT much easier to handle, as well as with less tension on the peripheral parts. Also, the donor site morbidity as mentioned makes the RFF less favorable, since a skin graft to close the donor site will always be needed. This affects the patient both aesthetically and physically, compared to the laxity of the wasted skin of hypopharyngeal cancer patients, which makes primary closure very attainable after ALT flap harvest.

Our second technical point is the use of chimeric ALT flap versus fasciocutaneous ALT flap. In this series there was no significant difference in the rate of fistula, but a significant difference was observed in the hospital stay, a mean of 33.1 days in the patients using chimeric ALT flaps compared to a mean of 41.1 days in fasciocutaneous ALT flap subjects. We think the benefit of the chimeric flap is due to the role of muscle in protection of the anastomosis and the decrease of the severity of the leak thereafter, in which this group of patients would end up with a minor leak compared to a major leak if we use a fasciocutaneous flap only. Moreover, the use of chimeric ALT flap, which could provide an additional volume to obliterate the dead space caused by lymph node neck dissection, decreases the possibility of fluid collection and fistula as well.

Despite advances in microsurgical techniques, pharyngoesophageal reconstruction after salvage laryngopharyngectomy remains challenging. The results of this retrospective analysis suggest that the flap choice and tailored flap design can influence anastomotic integrity. For pharyngoesophageal reconstruction using tubed skin flap, we continue to favor the ALT flap as it proves to be reliable and versatile. The thin and pliable radial forearm flap can be used for a partial defect. However, the more technical demand with the anterolateral thigh flap must be weighed against an easily harvested radial forearm flap.

## Methods

This study was approved by the ethics committee of Chang Gung Memorial Hospital (CGMH)– Linkou Medical Center. Informed consent was obtained from all patients and the investigation was performed in accordance with the approved guidelines. A retrospective review was conducted on patients who had circumferential defects and received ALT flap or radial forearm flap for pharyngoesophageal reconstruction after salvage laryngopharyngectomy when local recurrence was detected after CCRT between July 1994 and May 2011 at CGMH, Taiwan.

**Figure 1:**
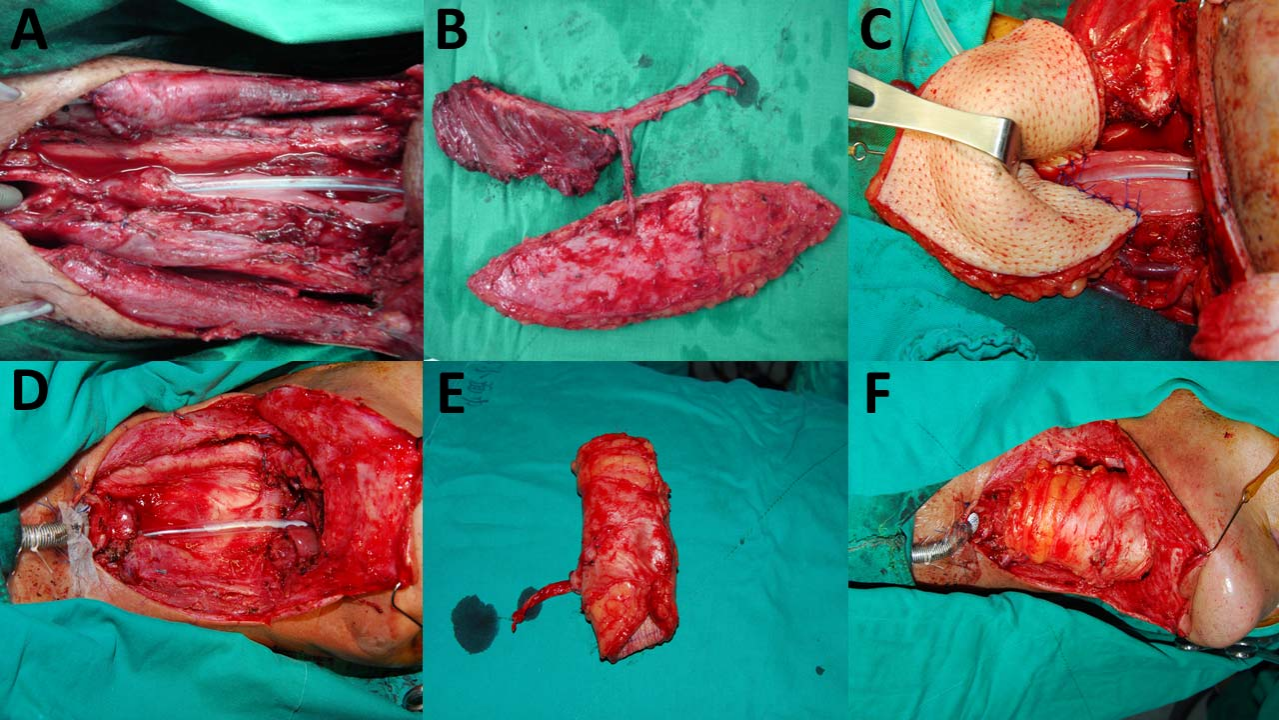
(A) A near-circumferential defect. (B) A chimeric anterolateral thigh (ALT) flap was composed of a skin paddle and a piece of vastus lateralis (VL) muscle. (C) The skin paddle was tubularized to form a neoesophagus and the VL muscle was used to increase tissue bulk and to obliterate the dead space. (D) A circumferential defect. After salvage laryngopharyngectomy with neck dissection, an “empty” neck was noted. (E) A tubed ALT free flap. (F) Immediate photograph after reconstruction.

An Allen test was mandatory to secure perfusion of the palmar arch by the ulnar artery alone. The free radial forearm flap was harvested using a suprafascial dissection technique^18^. The donor site was managed with either a split or full thickness skin graft. The design and harvest of the ALT flaps were performed as previously described^21^. When additional bulk was needed, vastus lateralis (VL) muscle was recruited in a chimeric fashion to provide the needed volume (Fig. 1). When anterior neck resurfacing was needed, given permissible anatomy, a separate skin island based on independent perforator was harvested for this purpose. Contralateral superior thyroid artery (STA) was preferentially used as the recipient artery as it tended to be minimally affected by concurrent chemoradiotherapy. Contralateral transverse cervical artery was used as an alternative when STA was not available. The skin flap was tubularized either by itself or in combination with residual pharyngeal mucosa to form a neoesophagus (Fig. 1). Laryngopharyngectomy and neck dissection were performed by head and surgeons, and the dissection of ALT flap was simultaneously performed by plastic surgeons. The harvest of radial forearm flap followed the completion of tumor ablative surgery. For buried flaps in the head and neck, direct monitoring of the skin flap can be difficult. For the ALT skin tube, the excess flap of the longitudinal end can be used as an external sentinel monitor for the buried part of the flap^28^.

All patients were admitted to a specialized microsurgery intensive care unit postoperatively for flap monitoring. Tube feeding was started on the first postoperative day. The patients were routinely transferred to regular wards on postoperative day 8. Liquid diet was started as soon as a contrast esophagogram on postoperative day 10 demonstrated intact anastomoses. The diet was advanced as tolerated. Fistula identified on the contrast esophagram were managed conservatively. Surgical treatments, including debridement and a second flap using the delto-pectoral flap or pectoralis flap as a patch plasty to close the fistula, were performed when the conservative management failed. Once fistula occurred, patients usually experienced a prolonged hospitalization. Patients were allowed to be discharged until fistula became healed or stable and they could take care of themselves.

All data were described as mean ± SD. Statistical analyses were performed using SAS software (version 9.1, SAS institute Inc., Cary, NC). All P values were 2-sided and statistical significance was accepted when *P* <0.05.

## Author Contributions

H.K.K. designed the study; K.P.C., C.H.W., S.Y.H. and B.H.S. collected and interpreted the data; H.K.K. and M.A. wrote the manuscript and prepared the figure; All authors reviewed the manuscript.

## Figures and Tables

**Table 1:** Patients' details between radial forearm flap (RFF) and anterolateral thigh (ALT) flap used for pharyngoesophageal reconstruction

	RFF	ALT	
	(n = 30)	(n = 43)	*p* value
**Period**	1994–2002	2001–2011	
**Age** (years)			
Mean ± SD (Range)	55.1 ± 10.0 (39–75)	56.6 ± 10.5 (36–82)	0.68
**Sex**			
Male	29	42	1.00
Female	1	1	
**Tumor**			
Hypopharynx	26	38	1.00
Larynx	3	4	1.00
Others	1	1	1.00
**Stage**			
II	3	4	1.00
III	7	9	1.00
IV	20	30	0.80
**OP time**			
Mean ± SD (Range)	705 ± 100.5 (420–900)	713.5 ± 120.3 (436–850)	0.54
**Flap size**			
Length (cm)			
Mean ± SD (Range)	10.2 ± 1.8(8–12)	12.2 ± 3.8 (8–16)	0.08
Width (cm)			
Mean ± SD (Range)	8.5 ± 1.3 (6–10)	10.6 ± 1.6 (6–12)	0.03*
**Donor site closure**			
Primary	0	34	<0.001*
Skin graft	30	9	
**Hospital stay**	48.6 ± 15.8 (27–80)	37.2 ± 12.5 (13–72)	0.03*
**In-hospital mortality**	1	1	1.00

*p <0.05.

**Table 2:** Patients' details between fasciocutaneous anterolateral thigh (ALT) flap and anterolateral thigh flap with chimeric vastus lateralis muscle for pharyngoesophageal reconstruction

	Fasciocutaneous ALT	Chimeric ALT	
	(*n* = 19)	(*n* = 24)	*p* value
**Age** (years)	54.6 ± 7.4 (42–68)	60.4 ± 10.9 (39–79)	0.11
Mean ± SD, range			
**Sex**			
Male	19	24	1.00
Female	0	0	
**Tumor**			
Hypopharynx	16	21	1.00
Larynx	3	2	0.64
Others	0	1	1.00
**Stage**			
II	2	1	0.58
III	2	5	0.44
IV	15	18	1.00
**OP time** (min)			
Mean ± SD (Range)	700.0 ± 132.3 (436–790)	732.8 ± 112.6 (570–850)	0.23
**Donor site closure**			
Primary	15	21	0.68
Skin graft	4	3	
**Hospital stay** (days)			
Mean ± SD, range	41.1 ± 14.3 (13–72)	33.1 ± 15.1 (15–70)	0.04*
**In-hospital mortality**	0	1	1.00

*p <0.05.

**Table 3:** Post-operative complications and outcome

	RFF^a^ (n = 30)	Fasciocutaneous	Chimeric		
		ALT^b^ (*n* = 19)	ALT (*n* = 24)	*P* value	
	*n* (%)	*n* (%)	*n* (%)	RFF *vs.* ALT	Fasciocutaneous *vs.* Chimeric
**Recipient site**					
Flap loss	1 (3.3)	1 (5.3)	0	1	0.44
Neck infection	18 (60.0)	10 (52.6)	6 (25)	0.06	0.11
Hematoma	3 (10.0)	1 (5.3)	1 (4.2)	0.40	1.00
Carotid artery blow out	1 (3.3)	2 (10.6)	0	1.00	0.19
Stricture	15 (50.0)	7 (36.8)	5 (20.8)	0.08	0.31
**Medical complications**					
Cardiac	1 (3.3)	1 (5.3)	0	1.00	0.44
Pulmonary	2 (6.6)	1 (5.3)	1 (4.2)	1.00	1.00
Renal	1 (3.3)	1 (5.3)	0	1.00	0.44
Hepatic	0	1 (5.3)	0	1.00	0.44
**Post-operative diet**^c^					
Soft	12 (41.3)	11 (61.1)	17 (70.8)	0.05*	0.53
Liquid	7 (24.1)	2 (11.1)	1 (4.2)	0.08	0.57
Partial TF^d^	7 (24.1)	2 (11.1)	4 (16.7)	0.36	0.68
Total TF^d^	3 (10.3)	3 (16.7)	2 (8.4)	1.00	0.64

^a^RFF, radial forearm flap;

^b^ALT, anterolateral thigh;

^c^one patient in the RFF and one patient in the fasciocutaneous ALT flap group were excluded;

^d^TF, tube feeding;

*p <0.05.

**Table 4:** Predictors of post-operative pharyngocutaneous fistula

Predictors	Pharyngocutaneous fistula (%)	*p* value
**Skin flap**		
aALT (*n* = 43)	13 (30.2)	0.03*
bRFF (*n* = 30)	17 (56.6)	
**ALT flap**		
Chimeric (*n* = 24)	6 (25)	0.51
Fasciocutaneous (*n* = 19)	7 (36.8)	

^a^ALT, anterolateral thigh;

^b^RFF, radial forearm flap.
